# Identification of a novel Golgi-localized putative glycosyltransferase protein in *Arabidopsis thaliana*

**DOI:** 10.5511/plantbiotechnology.23.1214a

**Published:** 2024-03-25

**Authors:** Natalia Rzepecka, Yoko Ito, Kei Yura, Emi Ito, Tomohiro Uemura

**Affiliations:** 1 Graduate School of Humanities and Sciences, Ochanomizu University, Tokyo 112-8610, Japan; 2 Institute for Human Life Science, Ochanomizu University, Tokyo 112-8610, Japan; 3 Natural Science Division, Faculty of Core Research, Ochanomizu University, Tokyo 112-8610, Japan; 4 Graduate School of Advanced Science and Engineering, Waseda University, Tokyo 169-8555, Japan; 5 Institute for Women’s Education in Science, Technology, Engineering, Arts and Mathematics, Ochanomizu University, Tokyo 112-8610, Japan

**Keywords:** glycosylation, Golgi apparatus, membrane trafficking, *trans*-Golgi network

## Abstract

SNAREs play an important role in the process of membrane trafficking. In the present research, we investigated subcellular localization of an uncharacterized *Arabidopsis thaliana* protein reported to interact with a *trans*-Golgi network-localized Qa-SNARE, SYNTAXIN OF PLANTS 43. Based on the similarity of its amino acid sequence to metazoan fucosyltransferases, we have named this novel protein AtGTLP (*Arabidopsis thaliana* **G**lycosyl**T**ransferase-**L**ike **P**rotein) and predicted that it should be a member of yet uncharacterized family of Arabidopsis fucosyltransferases, as it shows no significant sequence similarity to fucosyltransferases previously identified in Arabidopsis. AtGTLP is a membrane-anchored protein, which exhibits a type II-like topology, with a single transmembrane helix and a globular domain in the C-terminal part of its amino acid sequence. Colocalization data we collected suggest that AtGTLP should localize mainly to Golgi apparatus, especially to certain zones of *trans*-Golgi. As single *atgtlp−/−* mutants showed no obvious difference in phenotype (primary root length and fresh mass), AtGTLP and proteins related to AtGTLP with high similarity in amino acid sequences may have redundant functions.

## Introduction

An efficient and well-organized intracellular transport system is a basic requirement for life to exist, as many components necessary for metabolic processes, that take place inside or outside of a eukaryotic cell, are produced and subsequently modified in different cell compartments. The transport system responsible for delivering cargo collected from one compartment (that is: a membrane-enclosed organelle, such as endoplasmic reticulum, Golgi, or vacuole) to its next destination has been given the name of “membrane traffic”.

In the membrane traffic, macromolecules are packed as cargo into vesicles, which then carry the cargo to another organelle, plasma membrane or extracellular space. SNARE (soluble *N*-ethylmaleimide-sensitive factor attachment receptor) proteins, a family of membrane-anchored proteins comprising of more than 60 members in Arabidopsis (Sanderfoot 2007), play an important role in this transport system. Assisted by other molecules, such as Rab GTPases and tethering factors, SNAREs mediate fusion between the membrane of vesicle carrying the cargo and the membrane of the organelle the cargo is addressed to (Stenmark 2009). To facilitate the fusion, SNAREs form a complex comprising of four SNAREs—three Q-SNAREs (Qa-, Qb-, Qc-SNARE), which reside on the target membrane and are characterized by the presence of glutamine as the central amino acid in SNARE motif, in addition to one R-SNARE residing on the transport vesicle, with arginine as the central amino acid (Fasshauer et al. 1998). When a R-SNARE comes into contact with the three Q-SNAREs, first a link is formed between the vesicle and the target membrane, and subsequently, due to the change in conformation of the SNARE complex from “loose” to “tight” state, the vesicle is pulled toward the target membrane, allowing the two membranes to fuse together and the cargo to enter the lumen of the organelle (Jahn and Scheller 2006). To ensure the specificity of fusion, a large number of SNAREs localizes selectively to a specific cell compartment. This characteristic made it possible for those SNAREs to be successfully used as organelle markers in subcellular localization studies (Uemura et al. 2004).

Through their crucial role in membrane trafficking, members of SNARE family in plants were reported to be implicit in a variety of biological processes, ranging from growth to biotic/abiotic stress resistance. One group of SNAREs, that has been linked to secretion-dependent resistance against pathogens, is SYP4 (SYNTAXIN of PLANTs 4) group. SYP4 group comprises of three members (SYP41, SYP42 and SYP43) with partially overlapping functions, all localizing to the TGN (*trans*-Golgi network). *syp42−/−syp43−/−* double mutant exhibits phenotype of shorter roots, semi-dwarfism, and early senescence, in addition to increase in susceptibility to host-adapted virulent powdery fungus *Golovinomyces orontii* infection, when compared to wild type (Uemura et al. 2012).

A research focused on interactome of Qa-SNARE proteins in Arabidopsis reported a number of new interactions between specific SNARE proteins and numerous proteins of diverse functions, including novel proteins with unknown functions (Fujiwara et al. 2014). One such interaction was found between SYP43 and an uncharacterized protein, which we decided to name AtGTLP (*Arabidopsis thaliana* **G**lycosyl**T**ransferase-**L**ike **P**rotein) due to the similarity to glycosyltransferases in its amino acid sequence.

Glycosyltransferases (GTs) are a large family of enzymes, characterized by their ability to transfer sugar moieties to a specific acceptor substrate creating a glycosidic bond. GTs are known to produce a variety of glycoproteins and small sugar-containing molecules with diverse biological roles, but in plants, these enzymes are especially important for processing products of photosynthesis and for biosynthesis of cell-wall (Keegstra and Raikhel 2001). At present, 567 Arabidopsis proteins recorded in the Carbohydrate Active Enzyme Database (CAZy) (Cantarel et al. 2009; http://www.cazy.org/e1.html, accessed Nov 1, 2023) with ‘GlycosylTransferase’ annotation have been further classified into 45 of 117 GT families known today (including GT0 or GTnc, ‘non-classified’). Independently from the sequence-based classification implemented in CAZy database, GTs can also be categorized according to the type of sugar they use as a substrate. One such example is the superfamily of fucosyltransferases (FUTs), which utilize guanidine 5′-diphosphate-β-L-fucose (GDP-Fuc) as a substrate (Martinez-Duncker et al. 2003). In plants, macromolecules known to incorporate fucose in their structures are *N*-glycans, *O*-fucosylated proteins, as well as components of cell walls, such as xyloglucans, arabinogalactan proteins and pectic polysaccharides—rhamnogalacturonan I (RG-I) and rhamnogalacturonan II (RG-II) (Soto et al. 2019). Based on characteristics of FUTs that have been identified thus far (a majority of which are putative fucosyltransferases), it has been established that fucose is attached to each of these types of macromolecules by a specific FUT(s). As our knowledge about plant FUTs is still lacking, the full scope of their biological functions is, at present, not fully understood.

In the present study, we observed subcellular localization of AtGTLP by cross-pollinating a transgenic Arabidopsis line expressing AtGTLP-mGFP construct with organelle marker lines, as the first step to investigate the functions of AtGTLP.

## Materials and methods

### Plant material and growth conditions

For colocalization observations, Arabidopsis plants were grown in growth chamber, in 22°C in long-day light (16 h/8 h, L/D) conditions on MS medium w/vitamins (Duchefa Biochemie), containing 0.3% gellan gum (Wako) and 2% sucrose. pH was adjusted to 6.265. Fluorescence was observed in cells in the elongation zone of roots of 5-day-old seedlings.

For analysis of mutant phenotype, two T-DNA insertion lines SALK_025999 (*atgtlp-1*) and SALK_011654 (*atgtlp-2*) were used. Columbia-0 accession plants were used as a control. Growth conditions and medium were the same as for colocalization analysis. For statistical analysis of primary root length and fresh mass, 25 individuals (11-day-old) were used for each genotype.

### RNA extraction and RT-PCR

RNA was extracted from 10-day-old Arabidopsis seedling with RNeasy® Plant Mini Kit (Qiagen). 100 mg of fresh tissue was used for each sample. cDNA was synthesized using SuperScript™ III Reverse Transcriptase (ThermoFisher Scientific). 5 µg of the extracted RNA was added to each reaction as a template. The presence or lack of *AtGTLP* expression in mutant lines was confirmed by PCR reaction using ExTaq polymerase (Takara). 1 µg of cDNA was used as a template. Primers used for RT-PCR are listed in Supplementary Table S1.

### Gene cloning

A 5.1 kbp fragment of nucleotide sequence comprising of the native promoter and *AtGTLP* (*At2g04280*) gene sequence flanked by 5′ and 3′ UTRs was amplified using PrimeSTAR® GXL DNA Polymerase (Takara). The amplified sequence was subsequently subcloned into a pENTR™ vector using pENTR™/D-TOPO Vector Kit (ThermoFisher Scientific) for directional cloning. The vector was used to transform One Shot™ TOP10 Chemically Competent *Escherichia coli* (ThermoFisher Scientific).

Transformants were selected on solid LB medium with kanamycin 50 mg l^−1^ and cultured overnight in liquid LB with the same antibiotic concentration. Isolated plasmid was linearized and fused with monomeric GFP (mGFP) (Segami et al. 2014) gene sequence using In-Fusion HD Kit (Takara). Product of this reaction was used to transform competent *E. coli* cells and the transformants selection procedure was repeated. Isolated plasmid was used as the entry clone for pHGW vector (Karimi et al. 2002) as the destination vector in Gateway™ LR Clonase™ II reaction (ThermoFisher Scientific). *AtGTLP-mGFP* in pHGW construct was transformed into competent *E. coli* cells and transformant colonies were selected on solid LB medium with 100 mg l^−1^ spectinomycin.

For creation of mRFP-GOS12 Golgi marker line, translational fusion between *mRFP* (Campbell et al. 2002) and *GOS12* were generated by inserting cDNA encoding *mRFP* in front of the start codon of *GOS12* genomic sequence including 1.9 kb of the 5′-flanking sequence and 1.2 kb of the 3′-flanking sequence in pENTR™ vector using In-Fusion HD Kit. The resulted mRFP-GOS12 construct was subcloned into pGWB1 by LR Clonase™ II (Nakagawa et al. 2007).

Primers used for gene cloning and linearization are listed in Supplementary Table S1.

### Generation of Arabidopsis transformants

Col-0 plants were infected with *Agrobacterium tumefaciens* transformed with the prepared plasmid containing the *AtGTLP-mGFP* construct using flower dip method (Clough and Bent 1998). Collected seeds were sown on MS medium with 2% sucrose, 0.8% agar with 25 mg l^−1^ of hygromycin and 250 mg l^−1^ of claforan.

Selection of T_1_ lines with a single *AtGTLP-mGFP* construct was conducted by sowing T_2_ seeds on selective MS medium with hygromycin. Lines, for which we observed 3 : 1 segregation ratio of resistant to non-resistant plants, were selected as single-insertion lines.

### Confocal laser scanning microscopy observations

For subcellular localization observations of AtGTLP protein, AtGTLP-mGFP single-insertion lines were cross-pollinated with organelle markers lines. mRFP-VAMP721 (Uemura et al. 2019) and tagRFP-VAMP727 (Ebine et al. 2008) lines were used as markers for transport vesicle from the TGN to the cell plate/plasma membrane or to the vacuole, respectively. mRFP-SYP61 (Shimizu et al. 2021), mRFP-SYP43 (Asaoka et al. 2013), and VHAa1-mRFP (Dettmer et al. 2006) lines were used as TGN markers. ST-mRFP was used as a *trans*-Golgi marker (Uemura et al. 2012) and mRFP-GOS12 as a Golgi marker (in this study).

Colocalization observations were conducted using Zeiss LSM980 with Airyscan 2 confocal laser scanning microscope system equipped with Plan-Apochromat 63x/1.40 Oil DIC M27 immersion objective. Excitation laser wavelengths of 488 nm and 543 nm were used. Pinhole was set to 1AU.

### Colocalization analysis

The degree of colocalization of each organelle marker with AtGTLP-mGFP was assessed by calculating Pearson correlation coefficient (PCC) (Adler and Parmryd 2010; Dunn et al. 2011). To obtain PCC values, Coloc 2 plugin for Fiji/ImageJ software (Schindelin et al. 2012) was used. For each organelle marker, three 50 µm×50 µm images with 15 randomly selected regions of interest (ROIs), freehand-drawn around the observed dot-like structures, were used for analysis (45 ROIs total for each marker). Noise in the images was corrected using the ‘Smooth’ function of Fiji/ImageJ. Line fluorescence intensity plots were generated using RGB profiler plugin for Fiji/ImageJ.

## Results

### AtGTLP is a putative glycosyltransferase

**Figure 1:**
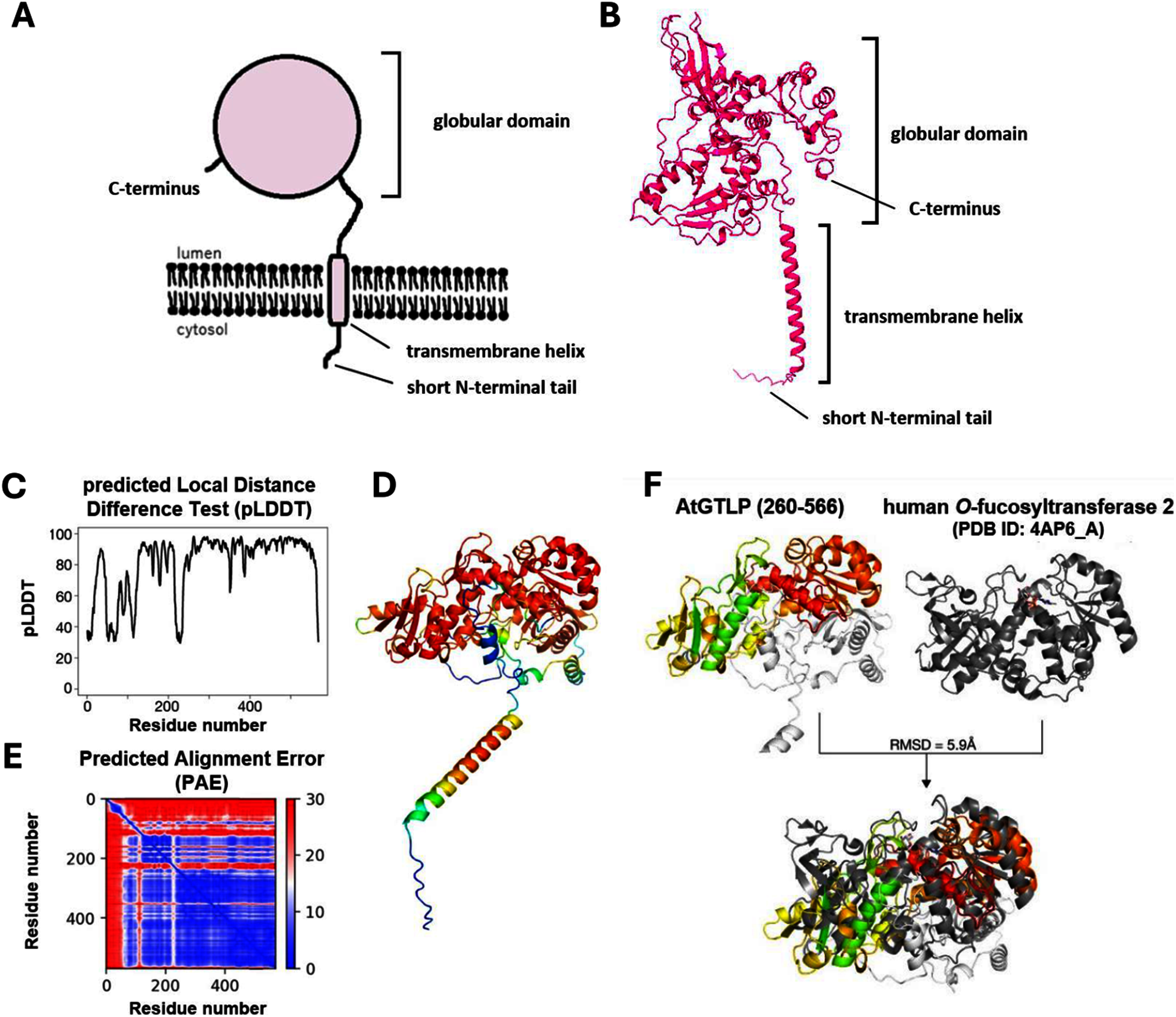
Figure 1. (A) Schematic model of AtGTLP topology and (B) AlphaFold2 prediction of the three-dimensional structure of AtGTLP. AtGTLP comprises of a short N-terminal tail, single transmembrane helix and globular domain in the C-terminal part of amino-acid sequence positioned on a short stalk (type II topology). (C) The prediction reliability described by predicted Local Distance Difference Test (pLDDT). Some of the regions in the N-terminal side have score less than 50 and the structures of those regions are less reliable. (D) Predicted three-dimensional structure of AtGTLP in pLDDT coloring. An α-helix at the bottom is the N-terminal side. Blue color indicates less reliable region and red color indicates region with high reliability. Note that the region with similar structure to *O*-fucosyltransferase 2 has high reliability. (E) Predicted Alignment Error (PAE). N-terminal side has high error (red), and C-terminal side has low error (blue). (F) Superposition between the three-dimensional structure of AtGTLP (from 260 to 566) and that of human protein *O*-fucosyltransferase 2 (PDB ID: 4AP6, A chain). The region in white in AtGTLP is the N-terminal region that was not included in the superposition. The gradation coloring scheme was employed from N-terminal to C-terminal side on the superposed region. Human *O*-fucosyltransferase 2 is colored in gray with GDP analogue in ball-and-stick model which suggests the active site of the protein. Root mean square deviation (RMSD) of the backbone is 5.9 Å. The similarity of the location of α-helices can be noticed.

AtGTLP is predicted to be a membrane-anchored protein comprising of 568 residues. DeepTMHMM (Hallgren et al. 2022; https://dtu.biolib.com/DeepTMHMM, accessed Nov 1, 2023) transmembrane topology prediction model for this protein indicates a presence of a single transmembrane helix near the N-terminus of AtGTLP protein (residues 24–44). The short N-terminal tail of the protein was predicted to localize on the cytosolic side of an organelle membrane, while residues 45–568 reside inside the lumen (Figure 1A). AlphaFold2 (Jumper et al. 2021) model of AtGTLP’s three-dimensional structure includes a globular domain of unknown function in the C-terminal part of the amino acid sequence (Figure 1B). The C-terminal part of the amino-acid sequence was modelled with high confidence, while N-terminal part had a lower reliability score (Figure 1C–E).

Analysis of AtGTLP’s amino acid sequence using Phyre^2^ server (Kelley et al. 2015; http://www.sbg.bio.ic.ac.uk/phyre2/html/page.cgi?id=index, accessed Nov 1, 2023) suggests a distant homology between the C-terminal domain of AtGTLP and human protein *O*-fucosyltransferase 2 (POFUT2; 19% of sequence identity, 99.2% confidence), protein *O*-fucosyltransferase 1 (POFUT1; 16% i.d., 98.9% confidence), as well as *C. elegans* protein *O*-fucosyltransferase 2 (CePOFUT2; 20% i.d., 98.9% confidence) and protein *O*-fucosyltransferase 1 (CePOFUT1; 18% i.d., 99.2% confidence). Superposition between AlphaFold2 model of C-terminal domain of AtGTLP and the top ranked template (human POFUT2; PDB ID: 4AP6, A chain) is shown in Figure 1F. Interestingly, among top ranked templates, we observed no plant GTs nor other types of plant enzymes. Moreover, although the Conserved Domain Database (Lu et al. 2020; https://www.ncbi.nlm.nih.gov/Structure/cdd/wrpsb.cgi, accessed Nov 1, 2023) recognizes the presence of ‘*O*-FUT-like’ domain in the C-terminal region of AtGTLP, when its amino acid sequence was used as a query in BLAST search against Arabidopsis proteome, none of the 38 proteins that have been characterized as members of Arabidopsis FUT family in literature (Smith et al. 2018) were present among the results. No other motif that could suggest possible functions of AtGTLP has been found to be present in its amino acid sequence. In the CAZy database, AtGTLP is currently classified as ‘GTnc’.

### AtGTLP is localized mainly on the *trans*-Golgi

**Figure 2:**
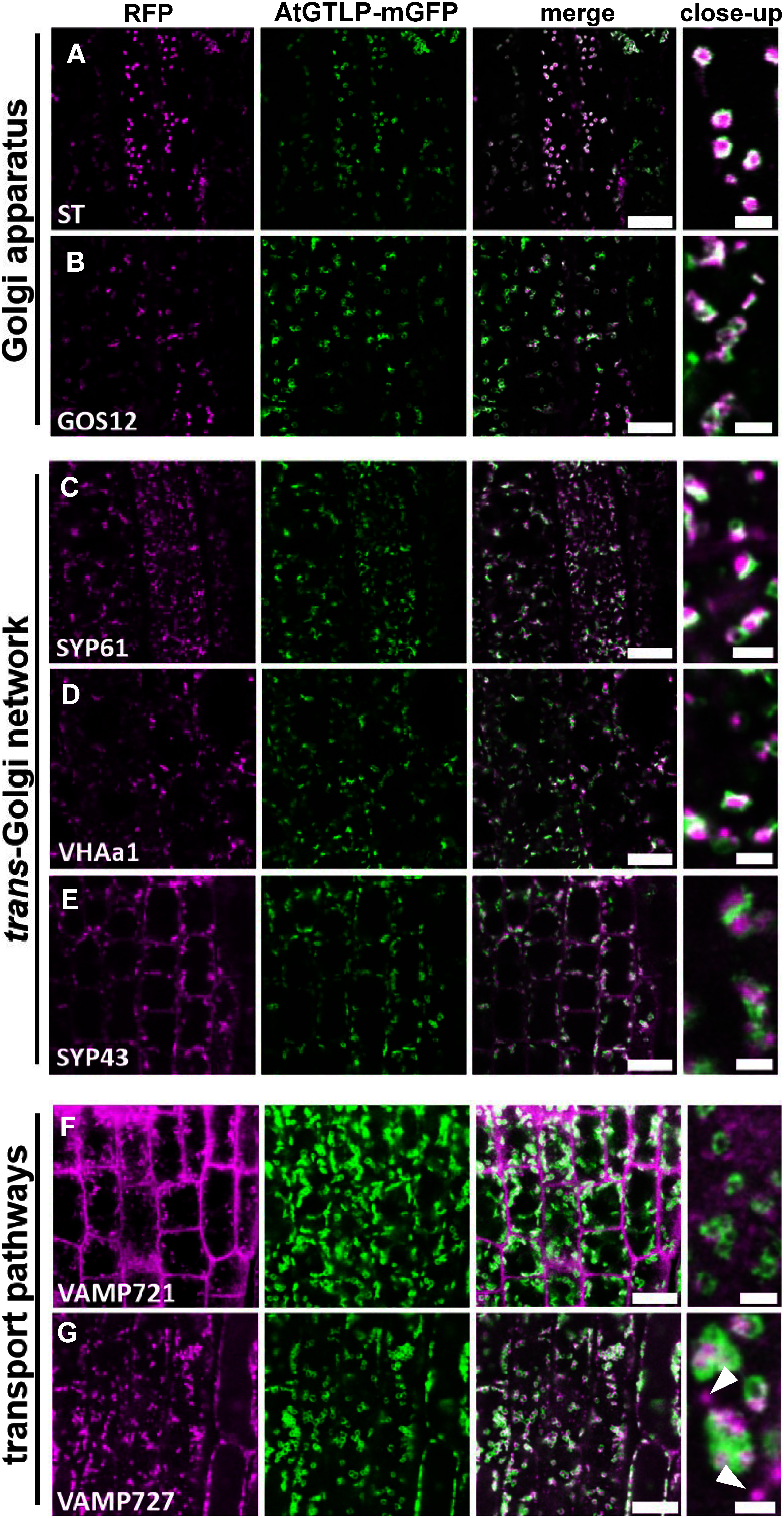
Figure 2. Subcellular localization of AtGTLP. Confocal laser scanning microscopy images of elongation zone cells of Arabidopsis seedling expressing AtGTLP-GFP construct, and organelle marker fused with RFP (A) ST-mRFP, (B) mRFP-GOS12, (C) mRFP-SYP61, (D) VHAa1-mRFP, (E) mRFP-SYP43, (F) mRFP-VAMP721, (G) tagRFP-VAMP727. Scale bar=10 μm. White arrow heads in (F) close up-dots of ‘free’ tagRFP-VAMP727. Close-up scale bar=2 μm.

To investigate the subcellular localization of AtGTLP, we established transgenic Arabidopsis expressing mGFP-tagged AtGTLP (AtGTLP-mGFP) under the control of native promoter. The fluorescence of AtGTLP-mGFP protein concentrated in dot- or ring-like structures scattered within the cell (Figure 2).

**Figure 3:**
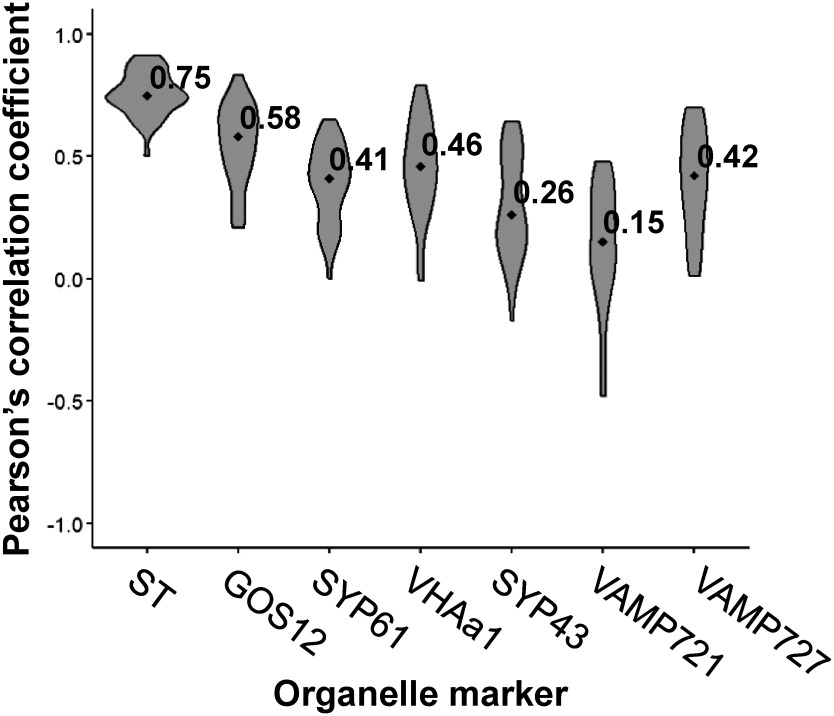
Figure 3. Colocalization of AtGTLP with organelle markers. As a mean of assessing the degree of colocalization, for each marker line expressing AtGTLP-mGFP and organelle marker fused with RFP Pearson’s correlation coefficient was calculated for 45 ROIs hand-drawn around clusters of dot-like structures. Three separate images were analyzed (15 ROIs/image) for each marker. Median is shown for each marker.

**Figure 4:**
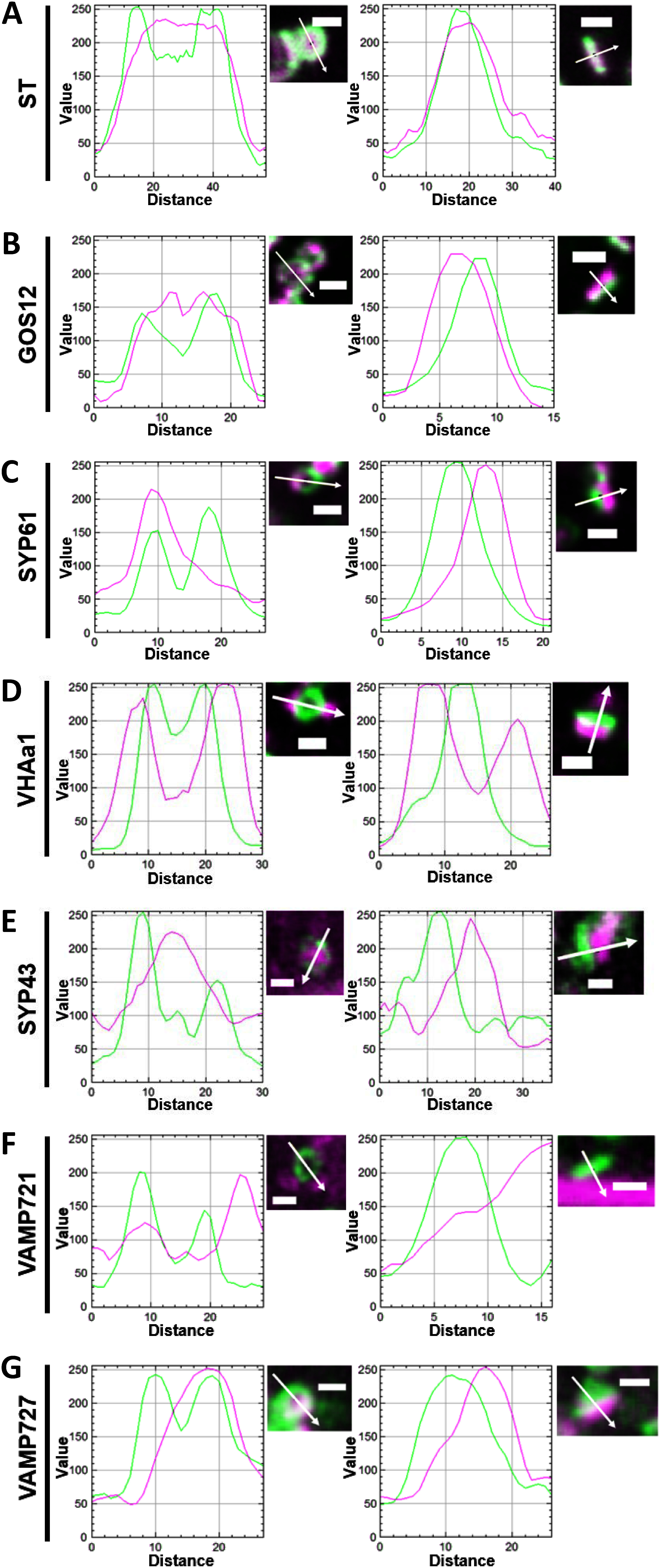
Figure 4. Line fluorescence intensity profile for (A) AtGTLP-mGFP and ST-mRFP, (B) AtGTLP-mGFP and mRFP-GOS12, (C) AtGTLP-mGFP and mRFP-SYP61, (D) AtGTLP-mGFP and VHAa1-mRFP; (E) AtGTLP-mGFP and mRFP-SYP43, (F) AtGTLP-mGFP and mRFP-VAMP721, (G) AtGTLP-mGFP and tagRFP-VAMP727 line. (A)–(G) left panel—en face orientation of AtGTLP ring; right panel—side orientation of AtGTLP ring. Intensity plot was generated along and in direction of the white arrow. Scale bar=1 μm.

In order to reveal which organelle those structures correspond to, we established double-visualization Arabidopsis lines expressing AtGTLP-mGFP with various RFP-tagged organelle markers. Among tested markers, the extent of colocalization of AtGTLP with *trans*-Golgi marker, ST (Boevink et al. 1998) was the highest and relatively consistent among all analyzed ROIs (Figure 3; PCC=0.5–0.91; median=0.75), suggesting that a significant population of AtGTLP localizes to *trans*-Golgi. Visual observations of colocalization of these two proteins show that while both seem to often localize to the same disk-like structures, patches of magenta-only and green-only fluorescence within those structures were also visible (Figure 2A, ‘close-up’). Based on this characteristic, we suspect that within *trans*-Golgi, AtGTLP segregates to specific zones only. As expected, relatively high PCCs were obtained also when colocalization with GOS12, a Golgi marker (Figure 2B; Uemura et al. 2004), was analyzed (Figure 3; 0.21–0.83, median=0.58). However, whereas for the AtGTLP-mGFP/ST-mRFP pair, the two peaks representing GFP and RFP in side-view line fluorescence profile of a single disk-like structure overlap almost entirely (Figure 4A), for the AtGTLP-mGFP/mRFP-GOS12 pair a slight shift between those two peaks was observed (Figure 4B).

Moreover, we found that in some of the analyzed ROIs, AtGTLP partially colocalizes with TGN markers, SYP61 (Figure 2C; Uemura et al. 2004) and VHAa1 (Figure 2D; Dettmer et al. 2006), whereas in other ROIs, PCC was close to zero (Figure 3; PCC=0.0–0.65; median=0.41 and PCC=−0.01–0.79; median=0.46, respectively). Side-view line fluorescence profiles for these two markers show a similar shift between the GFP and RFP peaks (Figure 4C, D). Interestingly, for the third analyzed TGN marker used in this study, SYP43 (Uemura et al. 2004), we observed that areas where the two colors of florescence overlap with each other, were scarcer (Figure 2E). Relatively low degree of colocalization for AtGTLP-mGFP/mRFP-SYP43 pair was also reflected in lower PCC values (Figure 3; −0.17–0.64, with median of 0.26) and line fluorescence profile, which suggest that SYP43 localizes to the center of AtGTLP rings (Figure 4E).

Taking into consideration the possibility that AtGTLP is transported within the cell as a cargo, two transport pathway markers—VAMP721, a marker of transport from the TGN to the cell plate and the plasma membrane, as well as VAMP727, a marker of vacuolar transport (Shimizu and Uemura 2022)—were also included in the study. In case of VAMP721, we observed very little overlap of GFP and RFP fluorescence (Figure 2F, Figure 4F), and low PCCs (Figure 3; −0.48–0.48, median=0.15), whereas colocalization between AtGTLP and VAMP727, similarly to SYP61 and VHAa1, was inconsistent across the analyzed ROIs (Figure 2G, Figure 3; PCC=0.01–0.7, median=0.42). Line fluorescence profiles for AtGTLP-GFP/tagRFP-VAMP727 pair also show a pattern similar to AtGTLP-GFP/mRFP-SYP61 pair (Figure 4G). When inspecting the degree of colocalization visually, we observed that the areas of overlapping fluorescence were present mainly within clusters of dot- or ring-like structures, which most likely correspond to Golgi/TGN. In addition to these clusters, ‘free’ dots of tagRFP-VAMP727 signal, where AtGTLP-mGFP was absent, could also be observed (Figure 2G, arrowheads).

### atgtlp mutants exhibit no obvious phenotypes

According to data available in eFP Browser (Klepikova Arabidopsis Atlas; Klepikova et al. 2016), *AtGTLP* is expressed in most parts of a plant, including roots and young leaves, with the exception of dry seeds.

**Figure 5:**
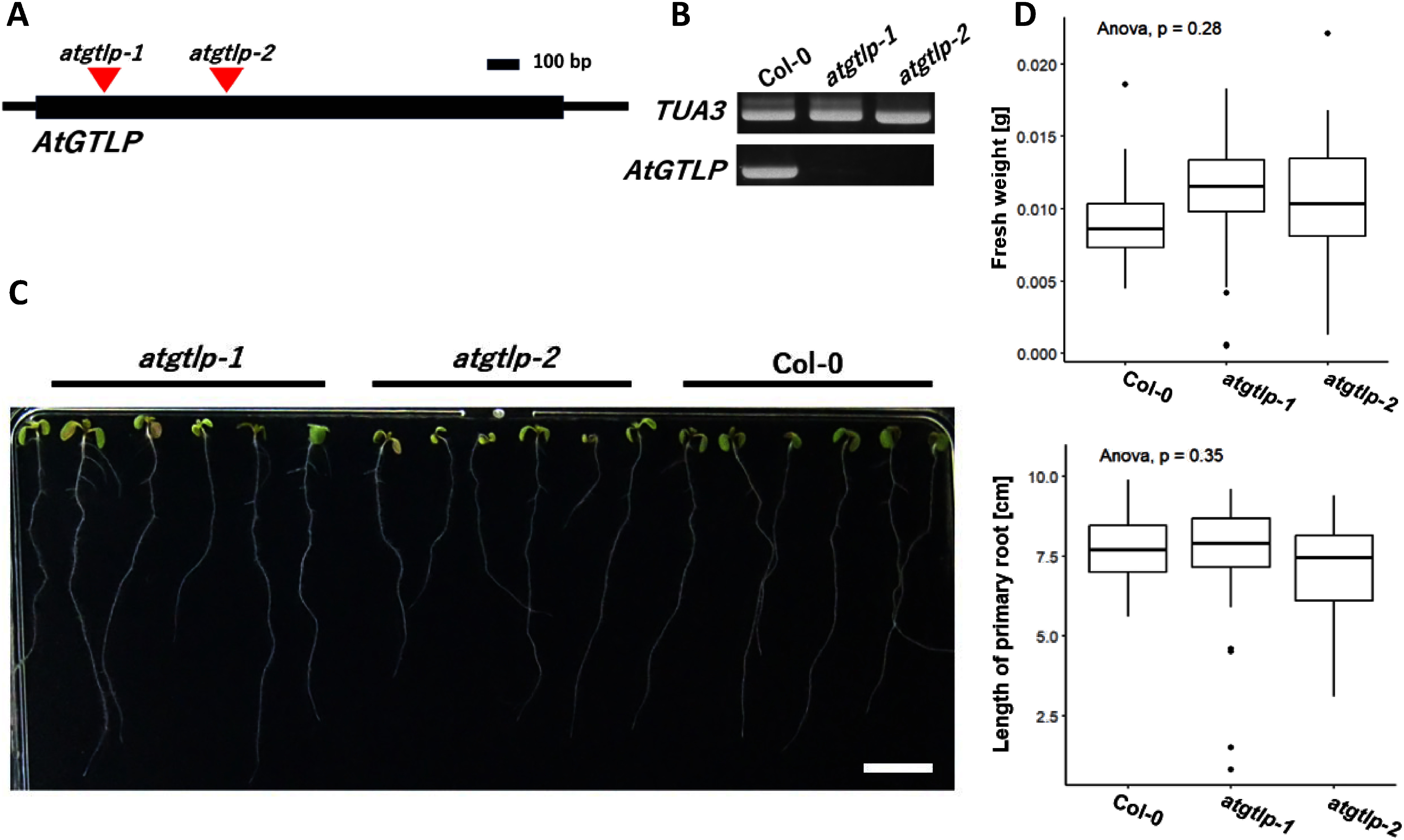
Figure 5. Characteristics of *atgtlp-1* and *atgtlp-2* single mutant lines. (A) T-DNA insertion sites inside nucleotide sequence of *AtGTLP* gene in *atgtlp-1* (SALK_025999) and *atgtlp-2* (SALK_011654) lines, (B) RT-PCR results, control—*TUA3* (tubulin), (C) Phenotype of *atgtlp-1* and *atgtlp-2* 8-day-old-seedlings no difference between Col-0 plants and mutants has been observed. Scale bar=1 cm. (D) Comparison of fresh weight and length of primary roots in Col-0 and *atgtlp-1*, *atgtlp-2* plants (*n*=25).

In both mutant lines used in this study, T-DNA insertion was confirmed to be present inside *AtGTLP*’s exon (Figure 5A). Although the results of RT-PCR confirmed that transcription of *AtGTLP* in both of the T-DNA lines is absent or reduced to levels undetectable with this method (Figure 5B), growth of mutant plants was comparable to wild type plants, and we could not observe any distinctive phenotype differences between *atgtlp-1* or *atgtlp-2* single mutants and wild-type plants in 8-day-old and 11-day-old seedlings (Figure 5C, D).

## Discussion

### *Trans*-Golgi localization of AtGTLP

In our subcellular localization study, we found that AtGTLP colocalized well with the *trans*-Golgi marker, ST, as well as with the Golgi marker, GOS12. Based on these results, we consider AtGTLP to be a resident Golgi protein, localizing mainly to *trans*-Golgi. Furthermore, the ring-like appearance of AtGTLP-mGFP fluorescence suggest that it localizes mainly to the rim of Golgi cisternae (Tie et al. 2018).

The results of this study showed that the predicted characteristics of AtGTLP match that of glycosyltransferases. In eukaryotic cells, glycosylation processes are commonly thought to occur mainly in ER and Golgi (Hansen et al. 2010). In plants, biosynthesis of many cell wall polysaccharides, another process that involves GTs, was also reported to take place in Golgi (Keegstra and Raikhel 2001; Saint-Jore-Dupas et al. 2004). Predicted topology model of AtGTLP suggests that it possesses a short cytoplasmic N-terminal tail and a single transmembrane helix, while its globular functional domain is positioned inside the lumen. Such topology, often described as ‘type II topology’ in literature, is common among Golgi-resident enzymes known to take part in protein glycosylation processes (Banfield 2011; Hansen et al. 2010; Welch and Munro 2019).

Our data suggest that whilst AtGTLP partially colocalized with TGN markers, SYP61 and VHAa1, and to a lesser extent, SYP43, in a portion of the analyzed ROIs, the number of ROIs, where the Pearson correlation coefficient was close to 0, which suggests no colocalization, is noteworthy. TGN is a vesicular-tubular compartment adjacent to the *trans* side of a Golgi apparatus, but generally considered to be a distinct organelle with functions different from the Golgi (Griffiths and Simons 1986). Indeed, more recently, existence of two types of TGN in plant cells, Golgi-associated TGN (GA-TGN) and Golgi-independent TGN (free-TGN/GI-TGN)—a subpopulation of the TGN that is physiologically separated from Golgi stacks, has been reported (Staehelin and Kang 2008; Uemura et al. 2014; Viotti et al. 2010). The general role of TGN is to receive proteins modified in Golgi and direct them either for vacuoles, the plasma membrane or for secretion (Griffiths and Simons 1986) and therefore, this organelle is considered to be an important hub in the membrane traffic system, where cargo proteins are sorted, before being dispatched for their next destination (Keller and Simons 1997). In addition to TGN-resident SNAREs, involved in transport, other TGN-localized proteins include lipid-processing enzymes, which help to pack cargo proteins into transport vesicles (Gu et al. 2001). Glycosylation is a sequential process, in which different types of sugar are added to glycans in a specific order, as the modified macromolecule travels from ER through Golgi apparatus to finally reach the TGN. In mammalian cells, some types of sugars, such as galactose and sialic acid, can be added to glycans in the TGN by TGN-localized GTs (Rabouille et al. 1995; Stanley 2011). In plants, however, there are no reports of glycotransferase activity in the TGN. Based on the collected data, we therefore propose that AtGTLP is a *trans*-Golgi-localized protein.

### AtGTLP—a novel fucosyltransferase?

Analysis of AtGTLP amino acid sequence has shown that its C-terminal domain bears resemblance to the conserved domain found in metazoan POFUTs, although no obvious sequence similarity was found between AtGTLP and the proteins which have been previously classified as members of FUT family in Arabidopsis. Although, at present, we lack evidence that would confirm GT or FUT activity in AtGTLP, we consider that this protein may potentially belong to a yet uncharacterized family of Arabidopsis proteins with either GT or FUT activity.

One interesting possibility that is worth considering is that AtGTLP is indeed a POFUT. Phylogenetic analyses report putative POFUTs in plants and suggest plant POFUTs are phylogenetically distant from POFUTs families in other organisms (POFUT1 and POFUT2 families) (Hansen et al. 2012; Soto et al. 2019). Interestingly however, recently described Arabidopsis putative POFUT, *O*-FUCOSYLTRANSFERASE1 (AtOFT1), implicit in pollen tube penetration through the stigma-style interface, was reported to diverge from this pattern. Phylogenetic analysis of *AtOFT1* has shown that it exhibits a relatively high sequence identity to members of POFUT1 family, compared to other putative POFUTs found in Arabidopsis (Smith et al. 2018). As in silico analysis suggests that AtGTLP may also be potentially related to metazoan POFUTs, the possibility that yet uncharacterized families of POFUTs, more related to metazoan POFUTs than previously reported POFUTs, exist in Arabidopsis genome is worth further examinations.

In our subcellular localization study, we found that AtGTLP localizes mainly to *trans*-Golgi. Human POFUT1 and POFUT2 localize to the ER (Luo and Haltiwanger 2005; Luo et al. 2006), where the *O*-glycosylation process for most proteins starts (Stanley 2011). Arabidopsis POFUT with confirmed activity, SPINDY, is known to be active in nucleus/cytoplasm (Bi et al. 2023; Kumar et al. 2023; Zentella et al. 2023). However, it is the only POFUT discovered thus far with this subcellular localization (Sun 2021). On the other hand, putative Arabidopsis POFUTs, such as AtOFT1 (Robichaux et al. 2023; Smith et al. 2018), as well as FRIABLE1 (Neumetzler et al. 2012) and ESMERALDA1 (Verger et al. 2016) were experimentally shown to localize to Golgi. In addition, α1,3-fucosylation in sycamore cells, as well as α1,4-fucosylation by FUT13 in Arabidopsis was shown to occur mainly in *trans*-Golgi cisternae (Fitchette-Lainé et al. 1994; Schoberer et al. 2013), suggesting that fucose is available at plant Golgi to be used as a substrate.

In relation to AtGTLP showing distant similarity to metazoan POFUTs, alternative possibility is that it may be a new type of plant fucosyltransferase. One type of macromolecules, which contain fucose are pectic polysaccharides—RG-I and RG-II. At present, biosynthesis of RG-I and RG-II is not fully understood, and no specific fucosyltransferase, which could be linked to synthesis of RG-I and RG-II side chains, has been identified (Soto et al. 2019; Voxeur et al. 2012), suggesting that yet uncharacterized type of fucosyltransferase is present in plants and responsible for this process.

GTs are a diverse family of enzymes and very few members of FUT family in Arabidopsis has been well-characterized thus far. Despite the fact that characteristics of AtGTLP, such as its *trans*-Golgi localization, type II topology and the presence of *O*-FUT-like conserved domain, seem to agree with the assumption that it is a member of the glycosyltransferase family, the data currently available are not enough to conclusively state that AtGTLP is a GT or a type of FUT, precisely. Under standard growth conditions, *atgtlp−/−* single mutant displayed phenotype indistinguishable from Col-0 plants and one possible explanation for this result is that AtGTLP and related proteins with high amino acid sequence similarity to AtGTLP might have redundant functions. Protein At4g12700, which shows a very high sequence similarity to AtGTLP is one potential candidate. Further research is needed to confirm the presence of glycosyltransferase/fucosyltranferase activity, as well as the biological role of AtGTLP.

## Abbreviations

FUT: fucosyltransferase
GT: glycosyltransferase
POFUT: protein *O*-fucosyltransferase
SNARE: soluble *N*-ethylmaleimide-sensitive factor attachment receptor
SYP: syntaxin of plants
TGN: *trans*-Golgi network

## Description of Supplementary Files

Supplementary Table S1. List of primers.
