# Supplementary material for: Identification of a novel Golgi-localized putative glycosyltransferase protein in Arabidopsis thaliana
Source: Plant Biotechnol (Tokyo). 2024 Mar 25;41(1):35–44. doi: 10.5511/plantbiotechnology.23.1214a (PMC11500582; doi:10.5511/plantbiotechnology.23.1214a)
Supplement: Supplementary Data [file plantbiotechnology-41-1-23.1214a-s001.pdf]

Supplementary Table S1.

Primer	Sequence
**RT-PCR**	
TUA3	forward 5'- GGA CAA GCT GGG ATC CAG G -3'
	reverse 5'- CGT CTC CAC CTT CAG CAC C -3'
AtGTLP	forward 5'- CGA TCT GCG ATT CGT GGA GG -3'
	reverse 5'- GAG CTT CAT GGG AGT GAC CC -3'
**Gene cloning**	
GOS12	forward 5'- CAC CGA AGA ATG AGT GAA AGA CAA GAG GT-3'
	reverse 5'- GTC TTA AAC CAA CGT AGA CGA AGA A-3'
AtGTLP	forward 5'- CACC ACG CTT GCA ATG AAT GGC ATC ACA -3'
	reverse 5'- GGG GAA GAG GAC GCG GCT TG -3'
mRFP	forward 5'- ATG GCC TCC TCC GAG GAC GT -3'
	reverse 5'- CAT GCC ACT ACC TCC GGC GCC GGT GGA GTG GCG GCC C -3'
mGFP	forward 5'- GGA GGT AGT GGC ATG GTG AGC AAG GGC GAG GA -3'
	reverse 5'- TTA CTT GTA CAG CTC GTC CAT GCC G -3'
**Linearization**	
GOS12	forward 5'- GGA GGT AGT GGC ATG ACA GAA TCG AGT CTG GAT CTG C-3'
	reverse 5'- GCC CTT GCT CAC CAT TTT CCG ATC TTC AAC AAT TCA GAT C-3'
AtGTLP	forward 5'- CAT GCC ACT ACC TCC GGA AGT TGC AGC ATT GCA GGT CCC A -3'
	reverse 5'- GAG CTG TAC AAG TAA TCT CTG TCA TAT TCC TCA TTT ATT T -3'
